# Effects of autoclaving and disinfection on 3D surgical guides using LCD technology for dental implant

**DOI:** 10.1186/s41205-024-00214-1

**Published:** 2024-04-24

**Authors:** Badreddine Labakoum, Amr Farhan, Lhoucine Ben Taleb, Azeddine Mouhsen, Aissam Lyazidi

**Affiliations:** 1 Radiation-Matter & Instrumentation Laboratory (RMI), Faculty of Science and Technology, University Hassan 1st, Settat, Morocco; 2 Health Sciences and Techniques Laboratory, Higher Institute of Health Sciences (ISSS), University Hassan 1st, Settat, Morocco

**Keywords:** 3D printing, Drill guide templates, Implant guide, LCD technology, Mechanical proprieties, Sterilization

## Abstract

**Background:**

Surgical guides can improve the precision of implant placement and minimize procedural errors and their related complications. This study aims to determine how different disinfection and sterilization methods affect the size changes of drill guide templates and the mechanical properties of 3D-printed surgical guides made with LCD technology.

**Methods:**

We produced a total of 100 samples. Forty surgical guides were fabricated to assess the implant drill guides’ surface and geometric properties. We subjected sixty samples to mechanical tests to analyze their tensile, flexural, and compressive properties. We classified the samples into four groups based on each analytical method: GC, which served as the control group; GA, which underwent autoclave sterilization at 121 °C (+ 1 bar, 20 min); GB, which underwent autoclave sterilization at 134 °C (+ 2 bar, 10 min); and GL, which underwent disinfection with 70% isopropyl alcohol for 20 min.

**Result:**

The results show that sterilization at 121 °C and 134 °C affects the mechanical and geometric characteristics of the surgical guides, while disinfection with 70% isopropyl alcohol gives better results.

**Conclusion:**

Our study of 3D-printed surgical guides using LCD technology found that sterilization at high temperatures affects the guides’ mechanical and geometric properties. Instead, disinfection with 70% isopropyl alcohol is recommended.

## Introduction

Dental implants resemble natural teeth regarding aesthetic appeal and oral functionality [1, 2]. Dental implants are generally used to replace missing teeth, the leading causes of which are tooth decay due to gum inflammation, the wrong root canal, infections, etc. Replacing missing teeth with a long-lasting dental implant is a sophisticated alternative.

In the past, dental implant surgery was only conducted by implant experts and consultants. However, it has become a routine treatment within the repertoire of modern dental professionals. The demand for clinical training has shown an upward trend with the growing prevalence of oral rehabilitation [3].

A practitioner must possess comprehensive knowledge and appropriate training to administer therapy and conduct repairs effectively. Nevertheless, a significant proportion of practitioners need more surgical experience, potentially increasing the likelihood of errors in the placement of implants and elevating the danger of encountering problems [4]. One key challenge usually encountered by practitioners is the optimal regulation of surgical osteotomies and implant sites. Incorrect placement of dental implants can significantly increase the complexity of the restoration process, leading to substantial increases in both financial costs and the time required [5]. The precision of implant placement and the thickness of the surrounding bone at the implantation site substantially influence an implant’s stability [6, 7].

Computer-assisted implant surgery is currently widely advocated due to its ability to minimize inaccuracies in the placement of implants [8]. Computer-guided implant surgery has been greatly influenced by the utilization of various technologies such as conical beam computed tomography (CBCT) for obtaining 3D images of the jaw bone, intra-oral scanners, computer-assisted design (CAD) software, and computer-aided manufacturing (CAM), commonly referred to as 3D prints [9–13]. The methodology relies on the transference of the virtual implant plan to the surgical site, facilitating accurate implant placement [14]. Surgical guides have the potential to enhance the precision of implant placement and reduce the occurrence of procedural errors and associated problems, contingent upon their accuracy and stability [15].

Multiple 3D printing methods can be utilized to fabricate surgical guides. These technologies facilitate the generation of three-dimensional models through the utilization of digital data [16]. In the 1980s, Charles Hull developed the first form of additive manufacturing called the stereolithography apparatus (SLA), which consists of selectively exposing a liquid resin point by point to a light source using a moving UV (ultraviolet) laser beam [17, 18]. Digital light processing (DLP) technology uses stationary projection UV light reflected by a million-pixel digital microscope device instead of a projector focal point to create precise objects with fine geometries [19]. The 3D printer market is moving to a new 3D printing technology called liquid crystal display (LCD). This new technique uses a series of LED (light-emitting diode) lights shining through an LCD screen that acts as a torch between the LED light and the bottom of the resin tank, allowing light to pass only according to specific shapes. These procedures consist of selectively exposing a liquid resin to a light source.

The precision of a surgical procedure can be compromised if a manufactured surgical guide undergoes deformation or modification during the disinfection or sterilization process with an autoclave at 121 °C for 20 min or 134 °C for 10 min [20]. Hence, the sterilized surgical guide must comply with specific dimensional and mechanical precision standards. Examining the impacts of these techniques on the mechanical characteristics and dimensional alterations of 3D printed materials, as well as their behavior pre- and post-sterilization and disinfection, has significant importance.

While numerous investigations have been conducted on the dimensional precision of 3D-printed surgical guides for implant placement, the impact of sterilization on the mechanical characteristics of such guides produced by LCD printing technology remains to be explored.

This research investigates the impact of various temperatures during sterilization and the influence of disinfection processes on the dimensional alterations and mechanical characteristics of surgical guides using a 3D-printed LCD. Additionally, it aims to identify the most effective sterilization/disinfection method that maintains the surgical guides’ clinical performance while preventing damage or deformation during their use.

## Methods and materials

An intraoral scan of the entire arch and a jaw CBCT scan is necessary to generate a virtual model. We used EXOCAD Galway 3.0 (Exocad GmbH, Darmstadt, Allemagne), which facilitated the process of digitally identifying the location of the implants and generating a virtual model of the surgical guides. Subsequently, the surgical guide’s design was exported as an STL (stereolithography) file and utilized as a template for the printing machine.

The comprehension of the mechanical response of a surgically guided complex geometric structure following thermal and chemical treatments cannot be adequately elucidated solely based on the geometric properties of surgical guides. In instances of this nature, it is vital to effectively compare load–displacement curves to define a guide’s mechanical properties. Conducting investigations on standardized specimens is essential to assessing the material’s mechanical properties mentioned above.

A total of 100 samples were produced. Forty surgical guides were manufactured to assess implant drilling guide templates’ surface and geometric properties. The remaining sixty samples were standard tests produced by the ASTM D638-14, ASTMD790-03, and ASTME384 standards; these standards respectively pertain to the tensile properties of plastics, the flexural properties of unreinforced and reinforced plastics, and the compressive properties of rigid plastics. The objective of analyzing these standard samples is to investigate the mechanical properties, namely the tensile, flexion, and compression behavior of materials employed in printing technology.

The samples were fabricated using LCD, specifically the Mars 2 PRO printer (Elegoo, Shenzhen, China). A resin known as “SG100 Surgical Guide” was used for printing. The samples were categorized into four groups, each consisting of five samples: GC, group of control; GA, subjected to autoclave sterilization at 121 °C (+ 1 bar, 20 min); GB, subjected to autoclave sterilization at 134 °C (+ 2 bar, 10 min); and GL, disinfected with 70% isopropyl alcohol for 20 min. The post-treatment procedure involved immersing the samples in 99% isopropanol alcohol for 5 min, allowing them to air-dry, and then subjecting them to photopolymerization at a wavelength of 405 nm and a temperature of 25 °C for 10 min.

### Standard tests on specimens

#### Flexural test

**Fig. 1:**
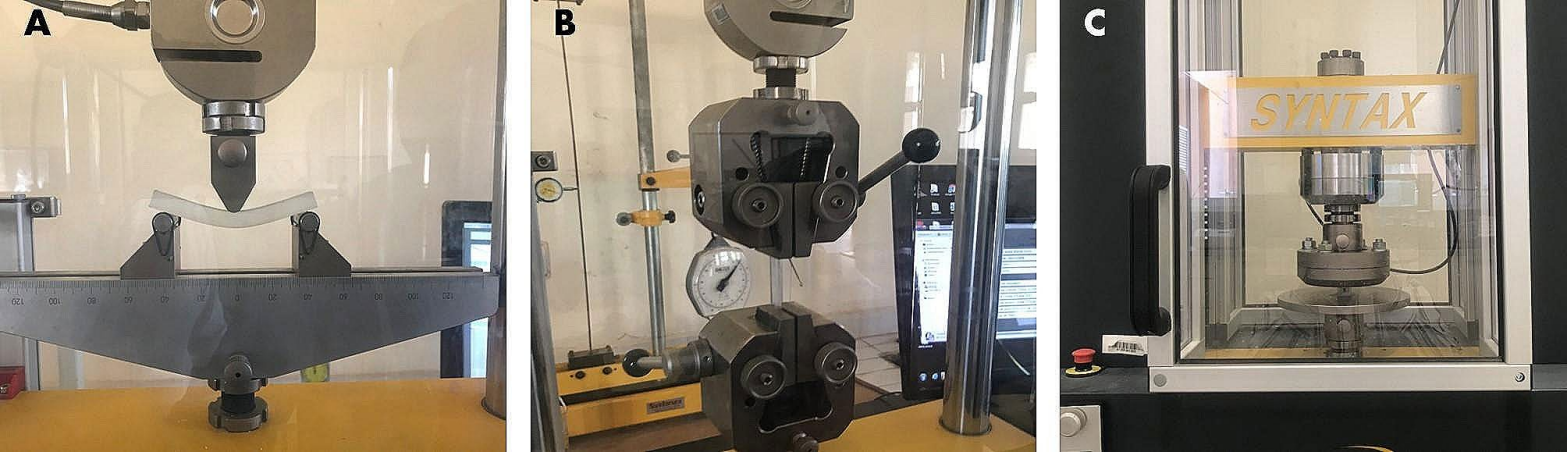
The specimens are undergoing testing to ascertain the mechanical properties of the materials under investigation: **A** three-point flexural test; **B** tensile test with regular Type V test; **C** compression test

A three-point flexural test was carried out using a universal test machine RP 25 ATF (3R, model RP 25, ATF, MONTAUBAN, FRANCE) to determine the material’s flexion strength (maximum flexural stress sustained by the specimen during the bending test), flexural strain and flexural modulus of elasticity. Each sample was placed precisely on two rounded, edge-shaped supports (a section of 2 mm). A third similar-shaped edge was positioned between the two supports, applying a continuous load of 0.1 mm/s until the sample detached (Fig. 1A). All values were calculated according to ASTMD79003, and the averages of five samples from each group were calculated.

#### Tensile test

A tensile test was conducted utilizing the universal RP 25 ATF using the type V tests outlined in ASTM D638-14 (Fig. 1B). The specimens were subjected to a loading rate of 0.1 mm/s. The materials’ tensile attributes were assessed, including tensile strength, tensile strain at maximum load, and tensile modulus of elasticity.

#### Compression test

The samples’ compression strength was evaluated using the SYNTAX Polyvalent universal test machine (3R, SYNTAC model, Montauban, France). The specimen was positioned between two flat surfaces (compression platens); a horizontal metal plate was then employed to exert a consistent compressive force of 0.1 mm/s onto the specimen until it reached the fracture point (Fig. 1C). The specimens underwent a concurrent loading condition consisting of compression load and flexion.

### Surgical guide examination

The measurements were performed to the submillimeter level in the places that should have been damaged the most. The critical area of the chirurgical guide is the drill guide templates that guide the surgical drill when creating the bone cavity for the implant during the implantation procedure. It is essential to know the location of the diameter holes before and after thermal and chemical treatments. The area of this section was measured at the level of the link section in the horizontal part of the drill guide templates using a stereomicroscope and alignment by scanning and surface analysis before and after sterilization and disinfection.

#### Stereomicroscopy examination

**Fig. 2:**
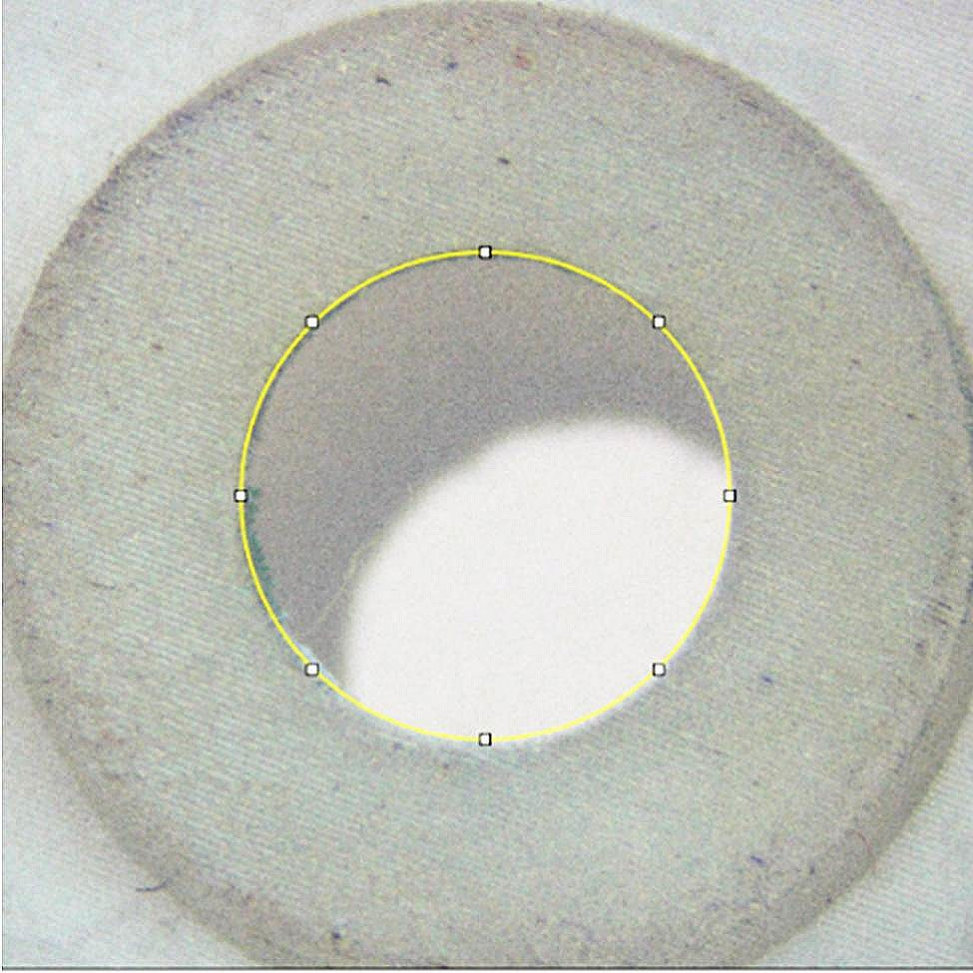
The provided image exhibits the measurements of the drill guide templates employed in surgical guides, as obtained by a stereomicroscope

To study the geometric surface of the measurements, we used the Leica m320 stereomicroscope (Leica, Heerbrugg, Switzerland) (Fig. 2). The stereomicroscope got pictures that were then used with the JMicroVision 1.3.4 program (JMi diagram, Geneva, Switzerland). Twenty guides were measured five times on each drill guide template before and after being disinfected and sterilized.

#### Image analysis and surface scanning of the surgical guide

After the manufacturing process of the surgical guide, an anti-reflection spray (Helling 3D Scan Spray; Laser Design, MN, USA) was applied to the drill guide template area. Subsequently, a three-dimensional dental scanner (MEDIT i500; MEDIT Corp, Republic of Korea) was utilized to scan the drill’s guide template surfaces, and the resulting 3D models in STL file format had an accuracy of 21.0 ± 1.48 μm. The mean thickness of the anti-reflection spray was 2.8 μm. The obtained STL files were compared to files scanned before and after disinfection or sterilization using surface alignment software linked to the scanning software (MeditDesign; MeditLink MEDIT Corp, Republic of Korea). Only the surface guidance sections that address the occlusal zones are utilized for accurate calculations between the reference and target data.

### Statistical analysis

The mean and standard deviation were employed to characterize the deviation measurement data derived from a range of mechanical experiments and stereomicroscopic observations. The accuracy was determined by calculating the average absolute disparity between the files before and after disinfection and sterilizing the surgical drill’s guide template dimensions using the root mean square (RMS). The precision is determined by applying the standard deviation (SD), which was utilized to compute the dimensional deviations in the surgical drill guide template size files before and after disinfection or sterilization [21]. The non-parametric Kruskal-Wallis’s test compared more than two numerical series devoid of a Gaussian distribution. The XLSTAT software (Addinsoft, Paris, France) was utilized for these computations. The significance criterion has been established as α = 0.05.

## Results

### Standard tests on specimens

#### Flexural test

Table 1 presents the values of the parameters of the flexural strength, flexural strain, and flexural modulus of elasticity of LCD printed samples for the four groups (GC-GA-GB-GL).

**Table 1:** The specimens’ mean and standard deviations of the measured parameters during the flexural test. *P*-values comparisons between control and disinfection/sterilization groups

Groups	Control (GC) Mean ± Std Dev	Autoclave 121 °C (GA) Mean ± Std Dev	*p*-value	Autoclave 134 °C (GB) Mean ± Std Dev	*p*-value	Disinfected (GL) Mean ± Std Dev	*p*-value
**Flexural Strength (MPa)**	58,9 ± 4,12	70,64 ± 7,71	0.258	74,24 ± 16,67	0.113	79,94 ± 11,55	0.042
**Flexural Strain %**	8,95 ± 0,35	3,95 ± 0,72	0.003	8,03 ± 0,78	0.308	7,35 ± 0,31	0.089
**Flexural Modulus of Elasticity (Mpa)**	575,11 ± 114,64	1804,05 ± 144	0.002	1031,61 ± 171,88	0.174	1082,26 ± 114,41	0.089

The analysis of group values revealed that the flexibility module (*p* = 0.002) and flexion elasticity values in the GA group increased significantly (*p* = 0.003) in comparison to the control group (GL). In contrast, the flexural strength values in the GB group didn’t differ significantly from those in the GL group (*p* > 0.05), whereas they did differ significantly from the GL group (*p* = 0.042).

#### Tensile test

Table 2 presents the tensile strain, tensile modulus of elasticity, and tensile strength values of LCD Printed samples for the four groups (GC-GA-GB-GL).

**Table 2:** The specimen’s mean and standard deviations of the measured parameters during the tensile test. *P*-values comparisons between control and disinfection/sterilization groups

Groups	Control (GC) Mean ± Std Dev	Autoclave 121 °C (GA) Mean ± Std Dev	*p*-value	Autoclave 134 °C (GB) Mean ± Std Dev	*p*-value	Disinfected (GL) Mean ± Std Dev	*p*-value
**Tensile Strength (MPa)**	36,07 ± 4,45	8,47 ± 0,79	0.013	11,55 ± 1,31	0.141	36,50 ± 1,30	0.91
**Tensile Strain %**	7,23 ± 2,31	5,48 ± 0,81	0.46	3,37 ± 0,42	0.008	4,74 ± 0,17	0.257
**Tensile Modulus of Elasticity (Mpa)**	522,72 ± 123,83	157,37 ± 30,74	0.042	344,54 ± 39,87	0.308	770,36 ± 19,61	0.308

The tensile strength (*p* = 0.033) and tensile modulus of elasticity (*p* = 0.042) values of the GA group revealed a GC group ratio that was statistically significant. The statistical analysis of the measured parameters didn’t identify any significant differences between the GC and GL categories (*p* > 0.05). Compared to the control group, the tensile strain values of the GB group increased significantly (*p* = 0.008).

#### Compression test

The values of compression strength, maximum compression load, and compression extension to maximum compression load of LCD printed samples for each group (GC-GA-GB-GL) are presented in Table 3.

**Table 3:** The specimens’ mean and standard deviations of the measured parameters during the compression test. *P*-values comparisons between control and disinfection/sterilization groups

Groups	Control (GC) Mean ± Std Dev	Autoclave 121 °C (GA) Mean ± Std Dev	*p*-value	Autoclave 134 °C (GB) Mean ± Std Dev	*p*-value	Disinfected (GL) Mean ± Std Dev	*p*-value
**Compressive Strength (MPa)**	42,67 ± 3,85	67,42 ± 3,85	0.089	74,28 ± 2,67	0.013	42,32 ± 1,68	0.91
**Compressive Extension at Maximum Compressive Load (mm)**	15,18 ± 0,02	15,37 ± 0,22	0.159	15,24 ± 0,26	0.388	15,10 ± 0,07	0.129
**Maximum** **Compressive Load (N)**	17067,71 ± 1540,8	26970,51 ± 1542,17	0.089	29711,71 ± 1080,07	0.013	16929,01 ± 674,34	0.91

The compression strength and maximum compression load showed significant values in the GB group compared to the GC group (*p* = 0.013). No significant differences were found (*p* > 0.05) in compression strength, compression extension at maximum compression, and maximum compression charge between each GA and GL group compared to the test group.

**Fig. 3:**
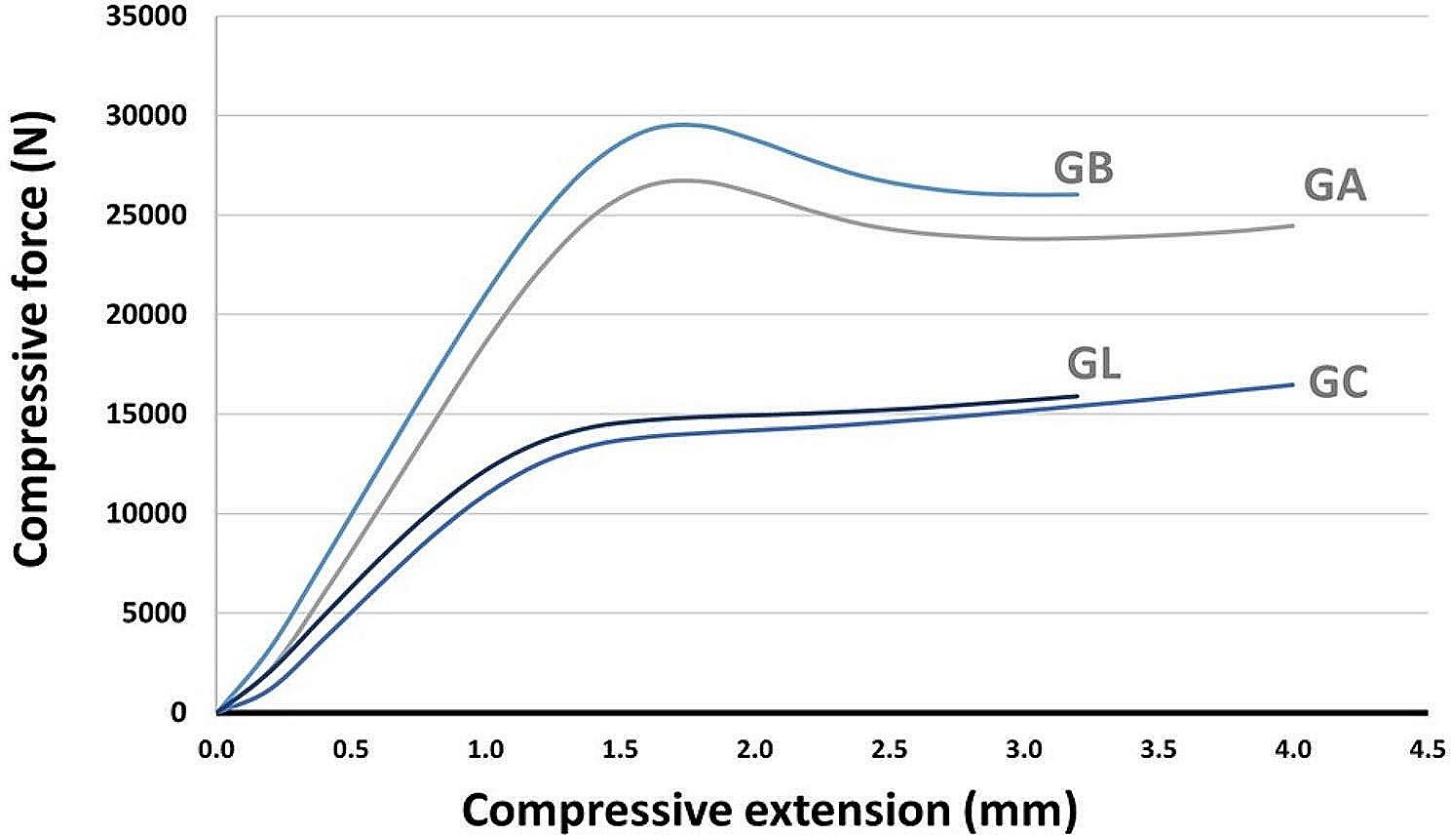
Surgical guides compressive load-deflection curves

The load-displacement curves for the surgical guide samples produced using LCD technology are compared in Fig. 3 across four groups: GC, GA, GB, and GL.

The acquired curves demonstrate a steady increase in the load force, followed by an abrupt interruption in the curve where the samples have undergone deformation. The test results indicate that the compression load experienced by the GA and GB groups was more remarkable than that of the control group. The control group exhibited a similar compression load and was closely aligned with the GL group. Hence, the GL group exhibited more substantial displacements than the GA and GB groups regarding the GC group.

### Surgical guide trials

#### Stereomicroscopy examination

**Fig. 4:**
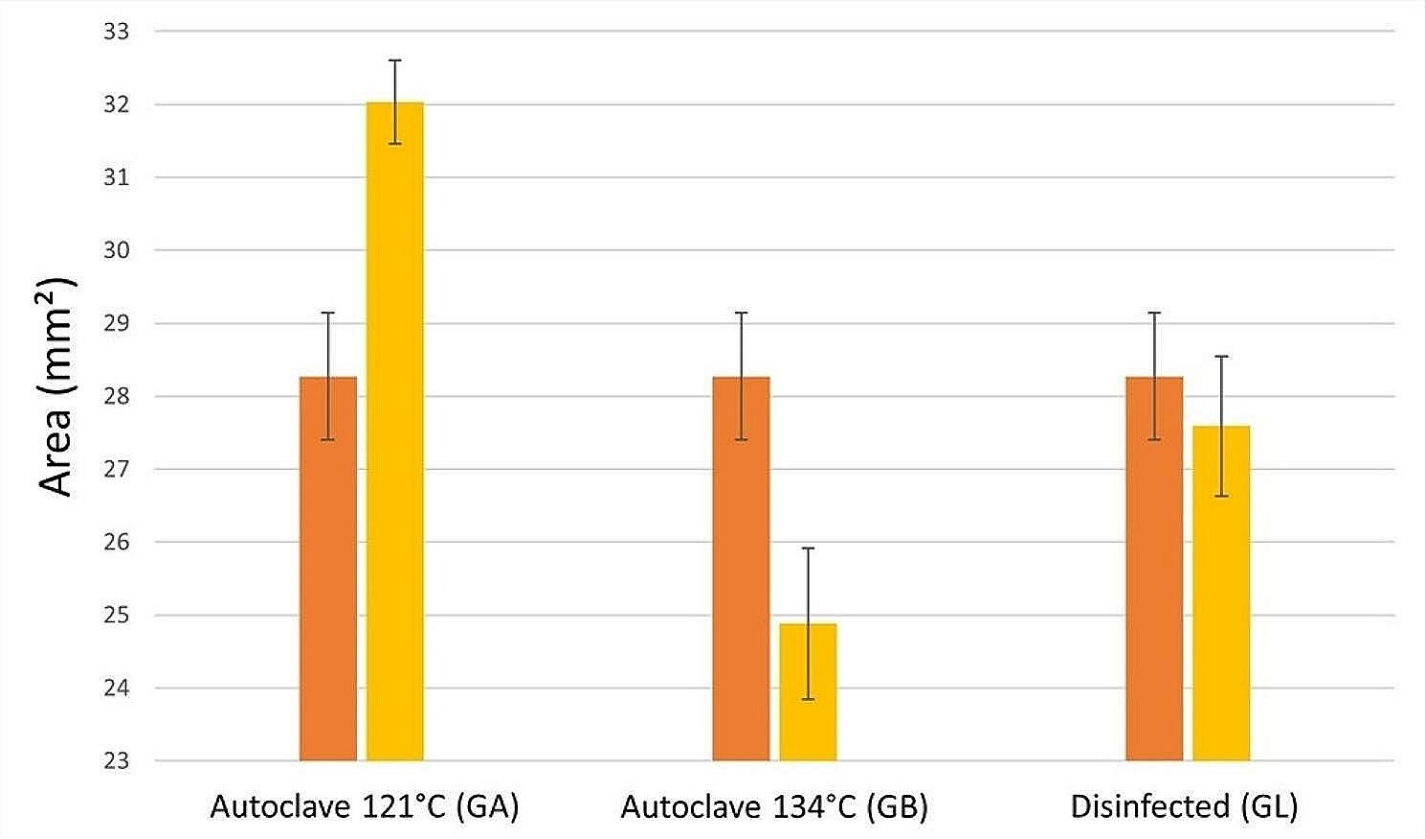
STEREOMICROSCOPY EXAMINATION. The geometric surface dimensions of drill guide templates were measured before and after disinfection and sterilizing procedures

The bar diagram (Fig. 4) displays the measured values of the geometric surface of the drill guide templates after disinfection and sterilization (yellow columns) and reference data (orange columns); each column shows the average and standard deviation of the measurements. These results indicate significant change and deformation in both steam sterilization groups (GA and GB), while 70% isopropyl alcohol disinfection indicates a negligible shift compared with the reference data.

#### Image analysis and surface scanning of the surgical guide

**Fig. 5:**
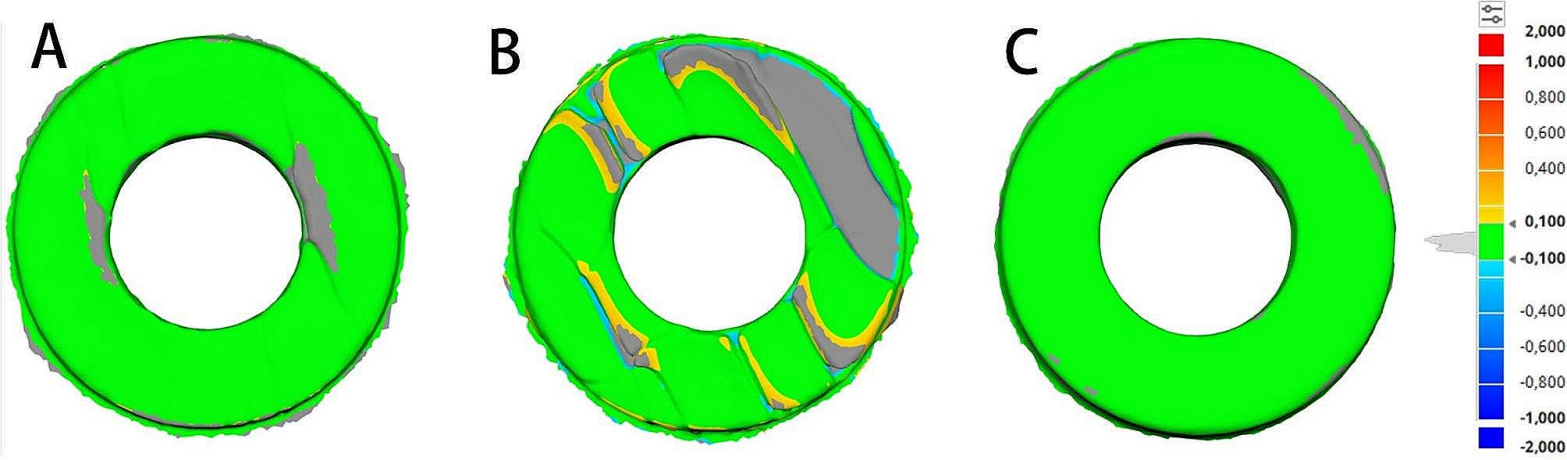
Colorimetric maps surface matching between the drill guide templates before and after disinfection or sterilization. **A** Sterilization autoclave 121 °C; **B** Sterilization autoclave 134 °C; **C** Alcohol Isopropyl for 20 min

The alignment of scanned surfaces with the dimensions of the drill guide templates in the surgical guides provides a surface color map based on the difference measured between the reference (before disinfection and sterilization) and target (after disinfection and sterilization) data scanned (Fig. 5).

The color green indicates a surface match within a tolerance of ± 0.1 mm. The blue zones, characterized by negative divergence, denote a smaller size of the target drill guide templates. Conversely, the yellow, orange, and red areas, characterized by positive divergence, indicate that the dimensions of the target drill guide templates are more significant than those of the reference surgical guide before and after sterilization and disinfection.

**Fig. 6:**
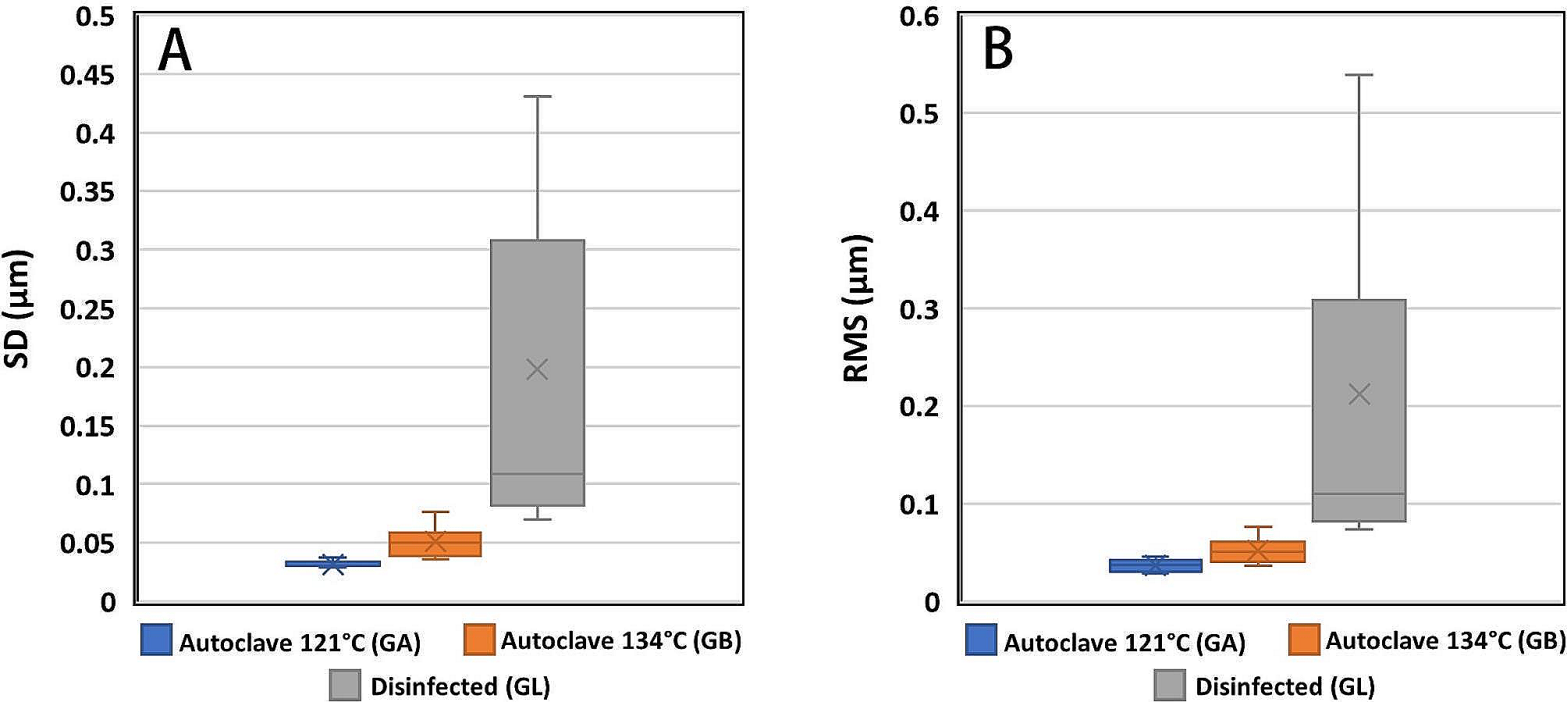
Boxplots of **A**, the precision (SD), and **B**, the trueness (RMS) of surface matching between drill guide templates before and after disinfection or sterilization, are depicted in a boxplot

The alignment between the GL and the GC donates the lowest SD value (0,0328, ± 0,0057 μm), followed by the steam sterilized group GA at 121° (0,0508 ± 1,0128 μm). For steam sterilization at 134°, the alignment of both GB and GC shows the most considerable SD value (0,1978 ± 0.1377 μm) (Fig. 6A).

The alignment between the GL and the GC donates the lowest RMS value (0,0365 ± 0,0064 μm), followed by the steam-sterilized group GA at 121° (0,0521 ± 0126 μm). For steam sterilization at 134°, the alignment of both GB and GC shows the most considerable RMS value (0,212 ± 0,1621 μm) (Fig. 6B).

GL revealed high trueness and precision in alignment with the GC, followed by the group GA, and GB revealed low trueness and precision.

The study results indicated that GL exhibited high accuracy and consistency in its alignment with the GC. In comparison, the group GA showed a relatively lower accuracy and consistency level, while GB exhibited the lowest levels of accuracy and consistency.

## Discussion

This study involved the production of 100 samples to assess the effects of disinfection and sterilizing processes on the dimensional changes of drill guide templates and the mechanical properties of surgical guides using 3D-printed LCD technology. The results show that sterilization at 121 °C and 134 °C affects the surgical guide’s mechanical and geometric characteristics. So, disinfection with 70% isopropyl alcohol was recommended.

A material’s mechanical properties and dimensional changes during a clinical procedure, such as sterilization and disinfection, are crucial for determining its behavior before clinical use, which may impact clinical performance [20, 22, 23]. Before and after chemical disinfection and autoclave sterilization, the mechanical properties and dimensional changes of surgical guides utilized in implant placement will be compared in this study.

Limited research exists on how chemical disinfectants and thermal sterilization affect the mechanical properties of surgical guides produced by new LCD printing technologies. Silvia et al. found that disinfection (4% Gigasept, autoclave sterilization at 121 °C and 134 °C) alters the mechanical properties of surgical guides produced through SLA and DLP printing [20]..

Smith et al. recommend disinfecting surgical guides with 70% ethanol for 15 min. Silvia et al. found that Gigasept 4% effectively disinfects both SLA and DLP printed surgical guides after 60 min of evaluation.

Torok et al. concluded that disinfection did not affect the parameters of 3D-printed surgical scales made with Objet MED 610 [23]. However, our study disinfected the samples with isopropyl alcohol for 20 min.

Based on the guidelines of the GC group, the tensile and compression test results of disinfected LCD printed samples did not show any statistically significant differences in the examined parameters. However, during the flexion test, some variations were observed in the flexural strength between disinfected samples and their base materials (GC). This discrepancy was caused by the higher radial rigidity of the sample disinfected with 70% isopropyl alcohol. This variation may be due to the printing process and the material used.

On the contrary, LCD-printed samples subjected to autoclave sterilization at 121 °C experienced a reduction in tensile load and deformation during bending. Moreover, the thermal effects that transpire during the sterilization process on the material’s behavior render the material more delicate. An additional manifestation of this consequence can be observed in the evolution of the elastic modulus (tensile and flexion), which deviates from the samples of the GC group that have not been subjected to thermal treatment. Conversely, the compression test becomes apparent when standard samples are compared. Meanwhile, Torok et al. reported that the material properties of the examined drilling dimensions were not significantly impacted by steam sterilization at 121 °C [23].

During autoclave sterilization at 134 °C, the maximum compressive load and compressive strength increased substantially compared to the control group. Additionally, standard tensile tests revealed only a minor deformation. Conversely, the flexion evaluations remain unaltered throughout the steam sterilization stage conducted at 134 °C. Consistent with the findings of Torok et al., autoclaving at 134 °C enhances the force of compression [23]. Silvia et al. observed that the mechanical properties of surgical guides were modified through thermal sterilization at 121 °C and 134 °C [20].

The samples that underwent autoclave sterilization exhibited a more vulnerable behavior in the compression load-displacement curves than the manufactured samples that had been disinfected. Thermal sterilization increased the rigidity of all specimens, and a higher sterilization temperature led to a more rigid guide.

The stereomicroscope analysis of our study revealed that steam sterilization (121 °C and 134 °C) causes a significant deformation, whereas 70% isopropyl alcohol disinfection results in a highly negligible and acceptable change. In contrast to Torok et al., they conclude that the stereomicroscope examination was not significantly altered or distorted by disinfection, plasma sterilization, or steam sterilization [23].

Alignment tests revealed that 70% of isopropyl alcohol-disinfested guides exhibited greater accuracy and precision, with no deformation observed during the disinfection process. Although steam sterilization yields lower accuracy and precision values, a notable deformation is observed in the autoclave set at 134 °C compared to the sterilization conducted at 121 °C. This result indicates that the geometry of the drill guide templates in the surgical guide is altered throughout the steam sterilization cycle.

During the sterilization and disinfection processes, the construction guidelines for surgical guides using LCD printing technology can cause substantial alterations to the material properties and guide geometry. Assessing the construction orientation may prove beneficial in acquiring data with greater precision.

The limitation of this study is that it examines the effects of sterilization and disinfection on specific methods without considering the broader range of techniques, such as plasma sterilization and 4% disinfection. Gigasept, with the increase in sample size, can also provide significant data for further studies. Comparative research is conducted to study the impact of sterilization and disinfection on surgical guides produced using various printing technologies, such as SLA and DLP. Understanding the effects of sterilization and disinfection on the mechanical and geometric features associated with each technique is crucial. It analyzes the geometric characteristics and dimensional accuracy of specimens using other methods.

## Conclusions

In this study, the impact of various temperatures during autoclave sterilization and disinfection with 70% isopropyl alcohol processes on the dimensional alterations and mechanical characteristics of 3D-printed LCD surgical guides was evaluated. Autoclave sterilization at 121 °C and 134 °C affects the surgical guide’s mechanical and geometric characteristics, whereas disinfection with isopropyl alcohol is appropriate for surgical guides.

## Data Availability

No datasets were generated or analysed during the current study.

## Abbreviations

CBCT: Conical beam computed tomography
CAD: Computer-assisted design
CAM: Computer-assisted manufacturing
SLA: Stereo-lithography apparatus
UV: Ultraviolet
DLP: Digital light processing
LCD: Liquid crystal display
LED: Light-emitting diode
STL: Stereolithography
RMS: Root mean square
SD: Standard deviation
